# Structural Racism and Obesity-Related Cancer Inequities in the United States: Challenges and Research Priorities

**DOI:** 10.3390/ijerph21081085

**Published:** 2024-08-17

**Authors:** Catherine M. Pichardo, Adaora Ezeani, Laura A. Dwyer, Anil Wali, Susan Czajkowski, Linda Nebeling, Tanya Agurs-Collins

**Affiliations:** 1 Behavioral Research Program, Division of Cancer Control and Population Sciences, National Cancer Institute, National Institutes of Health, Rockville, MD 20850, USAnebelinl@mail.nih.gov (L.N.);; 2 Cape Fox Facilities Services, Manassas, VA 20109, USA; 3 Community Outreach, Research, and Engagement Branch, Center for Cancer Health Equity, National Cancer Institute, National Institutes of Health, Rockville, MD 20850, USA

**Keywords:** racism, cancer, obesity, segregation, racial and ethnic health inequities

## Abstract

Structural racism has been identified as a fundamental cause of health disparities. For example, racial, ethnic, and economic neighborhood segregation; concentrated poverty; community disinvestment; and sociocultural context influence obesity and cancer disparities. Effects of structural racism are also evident through neighborhood obesogenic conditions such as limited access to affordable and healthy foods and physical activity opportunities within segregated communities that contribute to obesity and obesity-related cancer disparities. This article describes and expands on cross-cutting themes raised during a webinar held by the National Cancer Institute (NCI): (1) how structural factors, including neighborhood segregation and obesogenic conditions within racial and ethnic disadvantaged communities, influence disparities in the United States; (2) current research challenges and best ways to address them; and (3) selected priorities of the NCI aimed at addressing multilevel and intersecting factors that influence obesity-related cancer disparities. Further research is needed to understand how residential segregation and neighborhood obesogenic conditions influence cancer prevention and control across the continuum. Identifying the best approaches to address obesity and cancer disparities using social determinants of health framework and community-engaged approaches guided by a structural racism lens will allow researchers to move beyond individual-level approaches.

## 1. Introduction

Despite well-documented disparities in obesity and cancer, several research gaps remain in understanding how structural racism contributes to these disparities. To increase our understanding of the intersection of obesity, obesity-related cancer disparities, and pathophysiological and biological mechanisms within racially–ethnically diverse populations, the National Cancer Institute’s Obesity and Cancer Health Disparities Work Group held a webinar in April of 2023 entitled “The Role of Structural Racial/Ethnic Racism in the Intersection between Obesity and Cancer Health Disparities”. This paper expands on several cross-cutting themes that were raised during the webinar, particularly our understanding of multilevel determinants, including racial and ethnic segregation and related factors such as neighborhood obesogenic environment, biological mechanisms, and opportunities for primary to tertiary prevention. We define the neighborhood obesogenic environment as the sum of the influences of “socioeconomic and built environments [characteristics] relevant to energy imbalance that promote obesity”. This paper is not an exhaustive review of the literature, and several of the topics are discussed in more detail beyond the information provided by the webinar. Overall, we highlight structural racism and neighborhood segregation as risk factors for obesity and obesity-related cancers.

## 2. Structural Racism, Obesity, and Related Cancers

Structural racism and discrimination, defined as “macro-level conditions (e.g., residential segregation and institutional policies) that limit opportunities, resources, power, and well-being of individuals and populations based on race and ethnicity and other statuses”, is a fundamental cause of health disparities among underserved populations [1,2]. According to the ecosocial theory of disease distribution, the embodiment of structural racism and discrimination produces racial and ethnic disparities in health—mediated by physiology, behavior, and gene expression—driven by the interplay between environmental exposures, historical contexts, susceptibility, and resistance to disease [3]. In contrast, an ethnocentric perspective, which contributes to racism, does not consider how structural inequities impact obesity, obesity-related cancers, and other negative outcomes among underserved populations. To best address unequal access and disease burden among underserved populations, five domains that contribute to health and well-being have been identified: economic stability, education and access and quality, health care access and quality, neighborhood and built environment, and social and community context. Collectively, these domains are known as social determinants of health, the conditions in the environments where people are born, live, learn, work, play, worship, and age [4]. The ecosocial theory is an integrated approach that considers social determinants of health within a multilevel pathway lens to explain health disparities as mutable and embodied biological expressions of racism rather than exclusively resulting from differences in biological processes [5].

Research opportunities are needed to deepen the understanding of how forms of structural racism and inequities contribute to obesity and obesity-related cancers. Among the many forms of structural racism that impact obesity and cancer racial and ethnic disparities are neighborhood segregation, “the degree to which two or more groups live separately from one another in a geographic area” [6], deprivation, and the resulting neighborhood obesogenic conditions [7]. Jim Crow laws, discriminatory housing and financial practices, and large-scale public housing initiatives are posited to be significant drivers of segregation, intrinsically linked with racial and ethnic prejudice and discrimination, legitimized by White supremacy, and functioning to keep racial and ethnic minorities out of White spaces and limit residential mobility [2,8,9].

Racial and ethnic neighborhood segregation accounts for 70% of all racial and ethnic cancer disparities and is implicated in obesity disparities [10]. Among Blacks, studies have also linked neighborhood segregation to racial disparities in cancer incidence, breast cancer risk, ovarian cancer survival, colorectal cancer incidence, stage at diagnosis, mortality, and all-cause mortality [11]. While studies have examined current-day racial segregation and cancer using measures that capture racial and ethnic concentration and distribution across space, few studies have examined historical redlining and cancer. The consequences of historical redlining and current racial and ethnic segregation include, but are not limited to, concentrated poverty, isolation, limited access to health-promoting resources, environmental injustices, adverse neighborhood conditions, and disinvestment, which are strong drivers of cancer disparities [2].

On the other hand, social networks, social support, and social cohesion within segregated communities reinforce social and cultural norms that maintain positive health behaviors and have protective effects on some cancers [11,12]. For example, Hispanic/Latino segregation is generally associated with a lower incidence of breast and colorectal cancer and later stage at diagnosis, while mixed findings with mortality are reported [11]. Asian segregation is associated with lower incidence of breast and colorectal cancer [11]. However, these sociocultural resources (e.g., social support and community cohesion) may deplete over time, and increased gentrification can lead to heightened resegregation into more deprived areas and competition and decreased access to health-promoting resources such as affordable housing and medical services [13,14,15]. Depleted sociocultural capital over the long term within segregated and displaced communities has the potential to exacerbate cancer disparities. Future work should examine long-term exposure to segregation, particularly within the context of changing neighborhood environments.

## 3. Residential Segregation and Neighborhood Obesogenic Environment

Neighborhood obesogenic characteristics within segregated environments are strong drivers of obesity and cancer disparities. Limited access to cheap and healthy foods, medical resources, and physical activity opportunities, as well as increased exposure to environmental pollutants within segregated and disadvantaged communities interact to exacerbate obesity and obesity-related cancer disparities [7,16]. For example, The Clean Air Task Force and the National Association for the Advancement of Colored People in 2017 and Environmental Protection Agency in 2018 reported that over 1 million African Americans live within half a mile of an oil and gas operation [17]. Environmental pollutants are directly tied to breast cancer risk due to their ability to disrupt the endocrine system and epigenome-modifying effects [18]. Disadvantaged racial and ethnic communities have higher exposure to alcohol and energy-dense foods (e.g., fast food outlets) and have reduced access to healthy foods, which is directly tied to food insecurity among the general population and cancer survivors [7]. Residents of racially segregated and disadvantaged neighborhoods have lower availability of affordable healthy food (e.g., fruits and vegetables, quality milk, and produce) and reside further from full-service supermarkets compared with the residents of non-Hispanic White neighborhoods within the same socioeconomic classification [19].

Residents of segregated and deprived neighborhoods also have fewer opportunities to exercise due to structural inequities, which may, in turn, influence the intricate connections between energy balance, body size, and metabolic regulation [20]. Barriers to physical activity within impoverished neighborhoods include inequities in availability and/or quality of green spaces/nature, parks, recreational areas, physical activity facilities, cost and affordability of private recreational facilities, limited walkability (e.g., lack of sidewalks or poorly maintained sidewalks), bikeability, and safety concerns (e.g., proximity to highways with heavy traffic and high-crime areas) [21]. Segregated and deprived neighborhoods are at higher risk of crime, and residents of these neighborhoods have lower odds of physical activity likely due to perceived lack of safety [22]. Rectifying the obesogenic environment would require large-scale interventions that make healthy choices more convenient and affordable, reduce access to unhealthy products, and support community development. For example, to address stark differences in park access between communities in San Diego, CA, the city prioritized investments in long-ignored communities with few green spaces or parks to increase access to recreational activities. School programs [23], such as “Safe Routes to Schools”, that identify and create bicycle routes to schools and provide safety education for students have been shown to increase activity [24].

## 4. Metabolic Health and Biological Pathways

Adverse neighborhood conditions have been linked to poor metabolic health and other cancer-related biological mechanisms. There is evidence linking prolonged neighborhood deprivation and allostatic load, Ref. [25] the physiological burden of cumulative stress due to environmental challenges [26]. During allostasis, stress hormones (e.g., catecholamines and glucocorticoids) are released by the sympathetic nervous system, which, in turn, activates a host of molecular mechanisms, signaling, and epigenetic modifications implicated in cancer tumor initiation, development, and progression [18]. Chronic environmental stressors may induce these interrelated processes (e.g., mitochondrial dysfunction, obesity, insulin resistance, hyperglycemia, elevated lipid levels, and accelerated telomere attrition [27]) via increased oxidative stress and inflammatory events [18], all of which play a role in cancer development and progression [28,29]. Furthermore, socioeconomic disadvantage and neighborhood social environment, such as life and work pressure, poor lifestyle behaviors (e.g., reduced physical activity and lower quality diet), and psychological stress, can affect DNA methylation, a major epigenetic (method of) influencing gene expression [30,31]. These stressors can lead to activation of abnormal signaling pathways and increased gene mutation rates [31]. Subsequent gene expression of stress- and inflammation-related genes is a mechanism linked to cancer development and progression [31]. However, the literature remains limited in examining how neighborhood conditions are associated with cancer-related biological mechanisms, particularly over the life course, across generations, and in conjunction with obesity and race and ethnicity.

## 5. Future Work and Directions

Future work needs to disaggregate racial and ethnic subgroup data to better understand the heterogeneity and unique experiences with structural discrimination that result from individuals’ specific documentation status, migration experiences, and acculturative stress arising from different patterns of segregation. Racial and ethnic subgroups have differential availability of social capital and health resources, leading to differences in healthy lifestyles, metabolic health, and potentially varying cancer risks. Increasing our understanding of differential cancer and obesity risk by group characteristics will help tailor culturally appropriate targeted interventions and inform policy makers regarding allocation of resources [32]. Most of the empirical literature on behavioral interventions involves decreasing obesity among the general population, and the neighborhood literature heavily relies on the neighborhood socioeconomic status rather than other neighborhood factors. There is a need to understand the impacts of multilevel features of neighborhood environments on metabolic health, cancer recurrence, and mortality among racial and ethnic cancer survivors residing within segregated communities. Longitudinal, life course, and intergenerational research designs are needed to understand the effects of dynamic and changing neighborhoods (e.g., processes such as gentrification and urban-renewal), establish temporal associations, and identify dose–response relationships and critical windows for interventions [32].

Neighborhood improvements (e.g., increase in supermarket density and commercial physical activity facilities) that do not entail/address residential displacement/resegregation have the potential to significantly reduce obesity rates [33] and, in turn, cancer disparities. Potential solutions involve multilevel approaches that move beyond the individual and target policy changes both at the local and national level (e.g., inclusionary zoning and housing vouchers), community participation, culturally appropriate resources provided via organizations, and the strengthening of social capital resources within communities [34]. Equitable engagement of community stakeholders in nutrition and physical activity intervention and policy initiatives using a Community-Based Participatory Research (CBPR) framework has proven to be effective in identifying solutions to address the obesity epidemic [21]. CBPR provides community members and organizational representatives with decision-making power across all stages of the research process (i.e., identifying relevant research focus and questions, beneficial resources, study design, implementation, and sharing/interpretation of results) and requires direct benefits to communities being studied beyond knowledge generation [21,34,35].

Kumanyika et al. suggested interventions, with the goal to improve diet and physical activity and reduce obesity risk, that target (1) the built environment (e.g., neighborhood renewal programs and public–private partnerships supporting increased access to affordable healthy foods); (2) opportunities for physical activity (e.g., parks, recreational facilities, land use patterns, and socially and culturally centered urban design); (3) food environment (e.g., increasing healthy foods in neighborhood stores; healthy foods initiatives for mobile food vendors; and government nutrition assistance programs (e.g., nutrition program provisions designed for use at farmers’ markets)); and (4) social capital programs (e.g., community gardens) [36]. Williams and colleagues discussed successful policies and interventions with the goals of addressing high obesity rates in the United States; examples included The Parks After Dark program at the community level and the Sugar Sweetened Beverage Tax at the policy level [37]. Additionally, The National Cancer Institute is supporting interventions with the goal of addressing multilevel determinants of addressing obesity and related cancers. For example, The Promoting Weight-Loss in African American Cancer Survivors in the Deep South (DSNCARES) [38] intervention is a cluster randomized control clinical trial that addresses environmental weight loss barriers: (1) weight loss only, a 24-month trained lay provided weight loss program; (2) weight loss plus, the same program plus community-based weight management strategies; and (3) control, general cancer prevention education, among African-American breast, colon, or prostate cancer survivors and their family members residing in 9 rural communities in Alabama. The trained lay health educator arms components include behavioral dietary and physical activity techniques during in-person meetings, and the weight loss plus arm adds community-level strategies. This program has the potential for promising results given its multilevel approaches (e.g., individual, interpersonal, and environment/policy). Strengths include a CBPR approach that uses evidence-based community (farmers’ markets/gardens and improvement to walking trails) and culturally adapted strategies among hard-to-reach populations.

## 6. Conclusions and Future Research Priorities

The National Cancer Institute’s webinar, The Role of Structural Racial/Ethnic Racism in the Intersection between Obesity and Cancer Health Disparities, convened United States-based stakeholders to provide an overview of the role of structural racism in the intersection between obesity and obesity-related cancer disparities, describe pathophysiological and biological mechanisms, and identify evidence gaps and key next steps. During the webinar, experts identified the need for further understanding of multiple areas, including how residential segregation and adverse structural neighborhood conditions influence cancer prevention and control across the continuum. This paper expanded on some themes that emerged during the webinars by providing a brief overview of the literature regarding the impact of neighborhood segregation and related structural conditions on cancer outcomes, obesity, and related lifestyle factors. An important area for future work involves implementing multilevel and Community-Based Participatory Research (CBPR) to further understand and address obesity and cancer disparities, including cancer survivors, and investigate interactions between the neighborhood environment and biology. Framing disparities within multilevel frameworks will allow researchers to develop trials that use social determinants of health framework guided by a structural racism lens and move beyond individual willpower to manage weight and energy balance. The NCI’s programmatic activities focused on obesity-related cancer health disparities, and further research can be aimed at understanding the social and structural determinants of obesity and other modifiable risk factors that impact cancer risk and worsen cancer survival.

## Data Availability

https://cancercontrol.cancer.gov/brp/events/role-of-structural-racism-in-obesity-and-cancer-health-disparities (accessed on 14 February 2024).
